# Case Report: Paired vertex-occipital assessment reveals donor-area involvement in diffuse unpatterned alopecia

**DOI:** 10.3389/fmed.2026.1797275

**Published:** 2026-03-30

**Authors:** Yonglong Xu, Huaiwei Liu, Ying Xie, Shuying Lv, Meijiao Du, Lei Wang, Dingquan Yang

**Affiliations:** 1 Beijing University of Chinese Medicine, Beijing, China; 2 Department of Dermatology, The National Center for the Integration of Traditional Chinese and Western Medicine, China-Japan Friendship Hospital, Beijing, China

**Keywords:** androgenetic alopecia, diffuse unpatterned alopecia, donor area, follicular miniaturization, occipital scalp, scalp biopsy, trichoscopy

## Abstract

Diffuse unpatterned alopecia (DUPA) is an uncommon subtype within the androgenetic alopecia spectrum and is characterized by progressive diffuse thinning that may involve the entire scalp, including the occipital region, which is often presumed to represent a stable donor area for hair transplantation. We report two male patients with long-standing, progressive diffuse scalp thinning in whom paired vertex-occipital assessment demonstrated donor-area involvement. Quantitative trichoscopy showed increased hair-shaft diameter variability and elevated miniaturization in both the vertex and occiput, with consistently greater involvement in the vertex. Synchronous 4-mm punch biopsies from the vertex and occiput confirmed follicular miniaturization at both sites, with reduced terminal-to-vellus hair ratios and increased proportions of miniaturized follicles, supporting a diagnosis of DUPA and indicating the absence of a reliably stable donor zone. Both patients received medical therapy with oral finasteride and topical minoxidil. At 6 months, objective follow-up showed increased non-vellus hair density and reduced trichoscopic miniaturization in both scalp regions, consistent with short-term stabilization and partial improvement. This case series highlights an important diagnostic and management pitfall: if occipital sparing is assumed rather than verified, donor-area involvement may be overlooked and procedural counseling may be inappropriate. Paired vertex–occipital trichoscopy, with confirmatory biopsies when management decisions depend on donor stability, can improve diagnostic confidence, guide longitudinal monitoring, and inform medical and surgical counseling.

## 1 Introduction

Androgenetic alopecia (AGA) usually presents with regionally patterned thinning driven by androgen sensitivity (1). In many patients, the occipital scalp remains relatively preserved, which underpins the concept of a relatively stable donor area for hair transplantation (2, 3). Diffuse unpatterned alopecia (DUPA), however, presents with progressive diffuse thinning that may involve the entire scalp, including the occiput (4, 5). This pattern creates diagnostic uncertainty and complicates procedural planning because donor follicles may not be reliably spared.

Patients with diffuse non-scarring alopecia often report gradual loss of hair volume rather than abrupt shedding. On examination, thinning may appear relatively uniform across the frontal, vertex, temporal, and occipital scalp, and a hair-pull test may be negative. In this setting, the key diagnostic challenge is to distinguish DUPA from mimickers such as chronic telogen effluvium and alopecia areata incognita (6). Objective assessment of occipital involvement is clinically important because it may alter both diagnosis and donor-area counseling.

We report two male patients with diffuse scalp thinning in whom paired vertex–occipital assessment demonstrated donor- area involvement. Using quantitative trichoscopy combined with paired vertex and occipital scalp biopsies, we aimed to confirm pan-scalp follicular miniaturization and highlight the implications for medical management, longitudinal monitoring, and surgical counseling.

## 2 Clinical data

### 2.1 Patient information

Two male patients, aged 25 and 26 years, presented with long-standing progressive diffuse scalp thinning of 7 and 5 years' duration, respectively. Neither reported an abrupt shedding trigger such as febrile illness, major psychological stress, recent surgery, or rapid weight change. Case 1 had no major comorbidities, no relevant medication exposure, and no notable family history of alopecia. In Case 2, screening identified hyperuricemia and elevated homocysteine, without evidence of an alternative cause of diffuse alopecia.

### 2.2 Clinical findings

**Figure 1:**
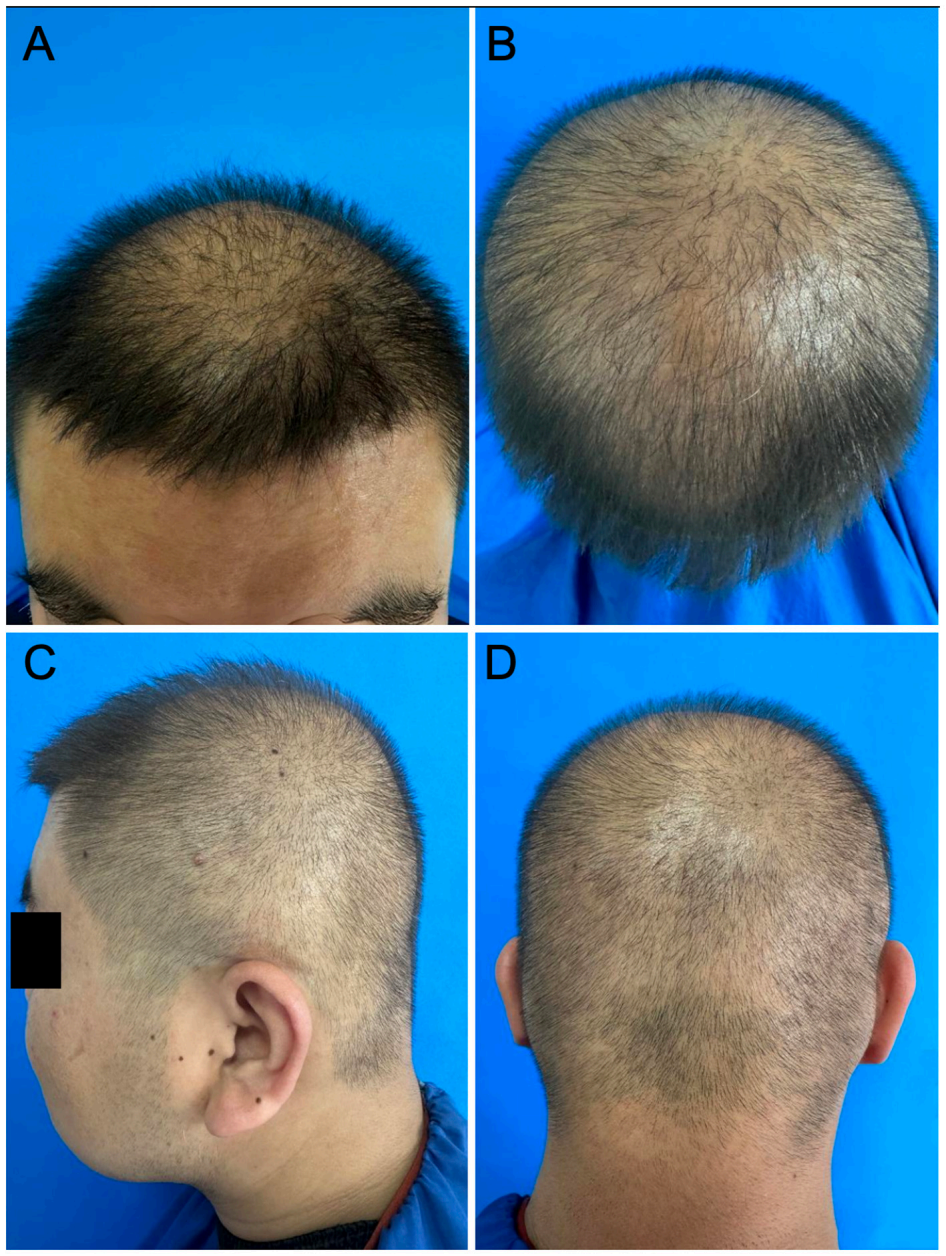
Clinical photographs of Case 1. **(A)** Frontal hairline. **(B)** Vertex. **(C)** Temporal region. **(D)** Occipital region. Diffuse thinning is evident across all scalp regions.

On examination, both patients showed diffuse, relatively uniform thinning involving the frontal scalp, vertex and parietal regions, temporal scalp, and occipital scalp, without a clearly spared donor zone. Hair-pull testing was negative in both cases, and there were no clinical signs of scarring alopecia, including loss of follicular openings, perifollicular scale, or erythema (Figure 1).

### 2.3 Diagnostic assessment

The differential diagnosis included classic AGA, chronic telogen effluvium, and alopecia areata incognita or diffuse alopecia areata. Screening laboratory evaluation included complete blood count, thyroid function testing, and iron metabolism markers. Results did not support systemic or nutritional triggers. Given the clinical overlap among diffuse non-scarring alopecias, objective assessment of miniaturization in the vertex and occiput was prioritized.

**Figure 2:**
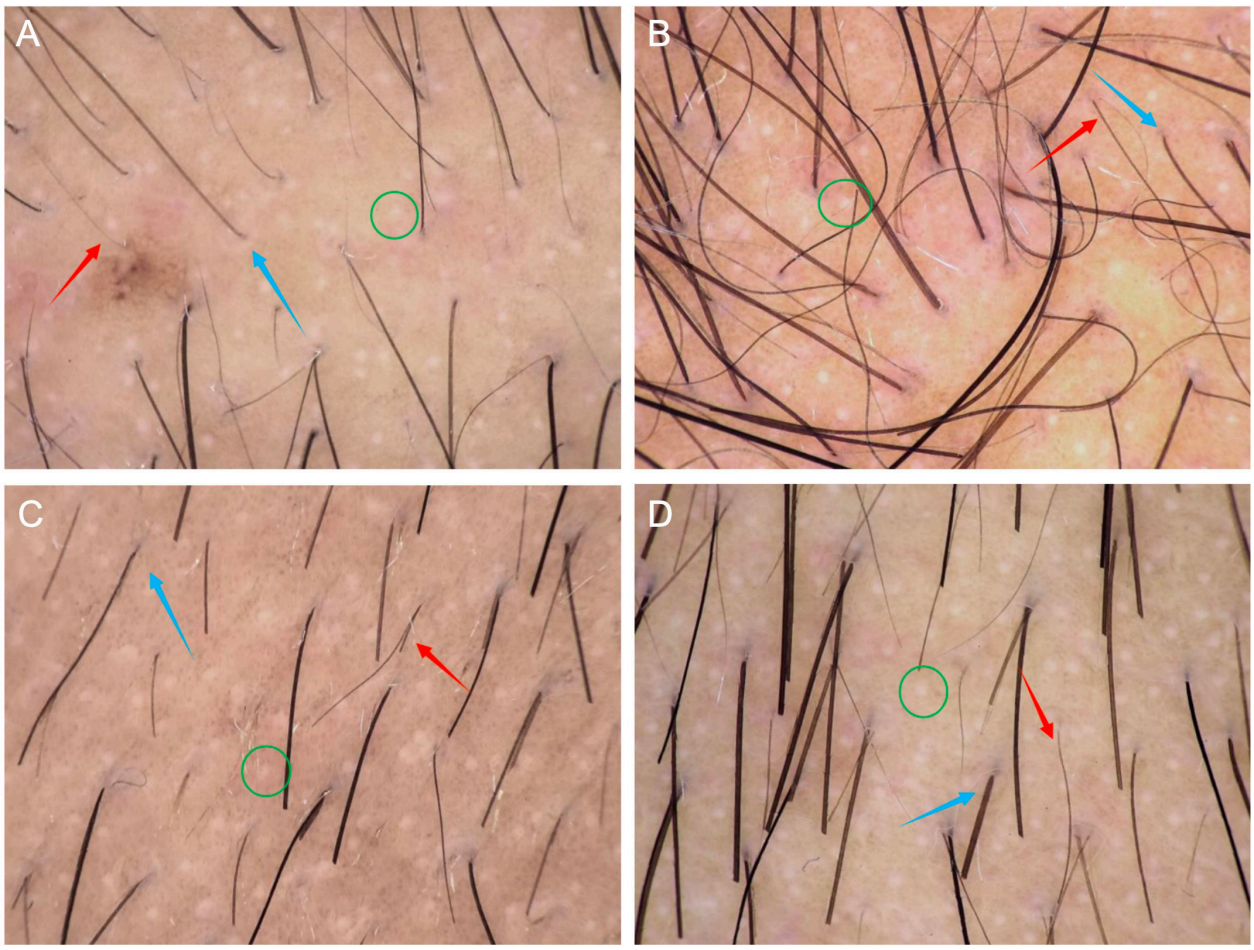
Trichoscopic findings in Case 1. **(A)** Vertex. **(B)** Whorl area. **(C)** Temporal region. **(D)** Occipital region. Trichoscopy shows increased hair shaft diameter variability (red arrow), reduced hair counts per follicular unit (blue arrow), and white dots (green circle) (Hongxin, polarized light, × 20).

Quantitative trichoscopy and digital hair imaging were performed as paired assessments of the vertex and occiput, the two regions most relevant to diagnostic confirmation and donor-area evaluation. A polarized 20 × imaging system was used for all examinations. Hair-shaft diameter variability and the proportion of miniaturized hairs were assessed in each region using at least three non-overlapping fields and values were averaged (Figure 2).

For density measurement, an approximately 1 cm^2^ target area within each region was trimmed to 0.5 to 1.0 mm following a standardized preparation protocol for digital hair imaging. High-resolution images were obtained and the number of non-vellus hairs within the target area was counted and reported as non-vellus hair density (hairs/cm^2^). In both patients, trichoscopy demonstrated increased diameter variability and elevated miniaturization in both the vertex and occiput, with consistently greater involvement in the vertex. Detailed quantitative findings are summarized in Table 1.

**Table 1:** Paired vertex and occipital trichoscopic and histopathologic findings in two patients.

Case	Site	Diameter variability at baseline, %	Miniaturization at baseline, %	Miniaturization at 6 months, %	Non-vellus density at baseline, hairs/cm^2^	Non-vellus density at 6 months, hairs/cm^2^	Terminal-to-vellus ratio at baseline	Miniaturized follicles at baseline, %	Inflammation grade at baseline, 0–3	Fibrosis grade at baseline, 0–3
1	Vertex	31.7	44.3	34.8	77.8	92.7	2.6:1	48.2	1	1
	Occiput	28.1	35.6	30.1	88.6	96.8	3.0:1	40.5	0	1
2	Vertex	29.9	40.6	34.2	82.4	95.3	2.8:1	43.7	1	0
	Occiput	25.7	32.7	28.0	90.2	100.6	3.1:1	36.2	1	1

**Figure 3:**
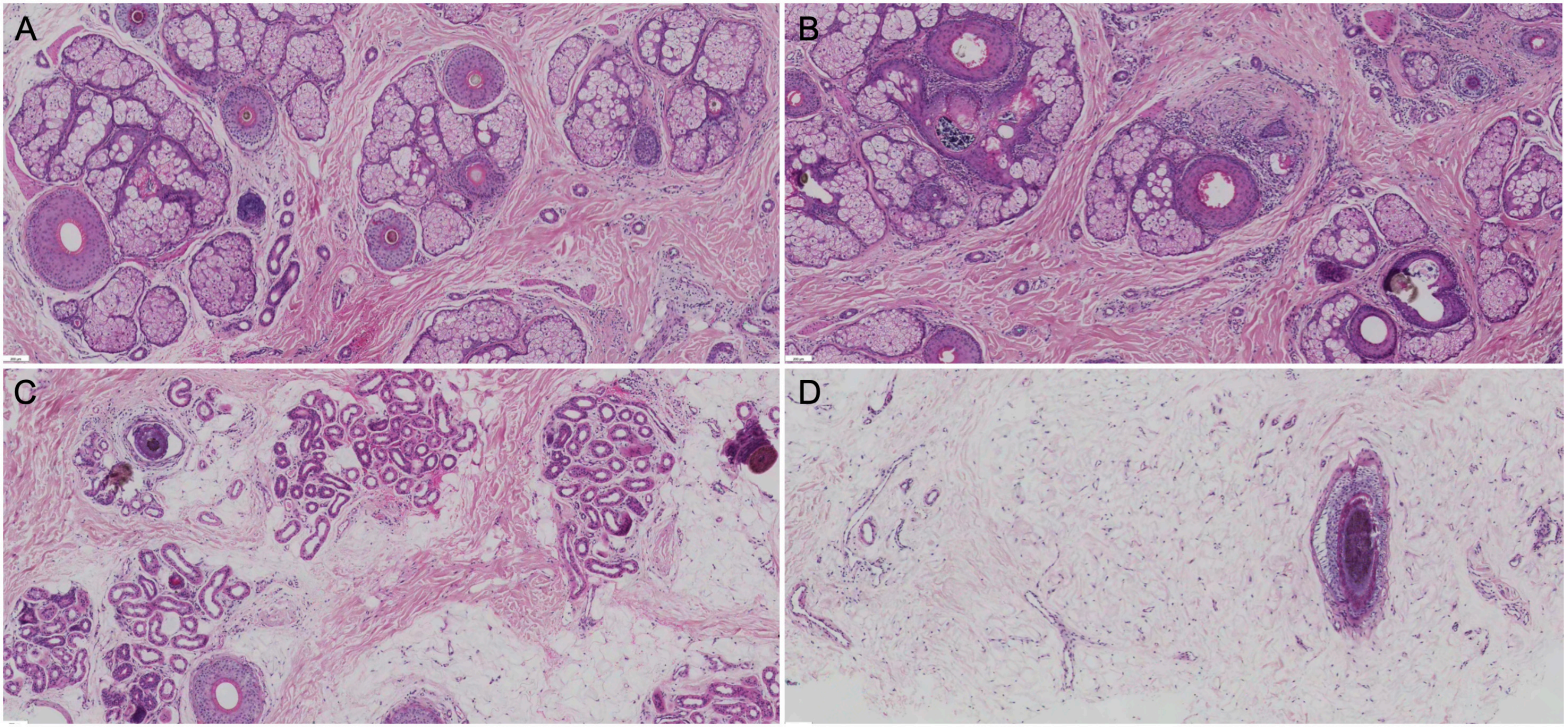
Histopathologic findings in Case 1. **(A)** Vertex scalp, longitudinal section, showing follicular miniaturization with reduced follicular density. **(B)** Vertex scalp, longitudinal section, showing mild perifollicular lymphocytic infiltration. **(C)** Occipital scalp, longitudinal section, showing follicular miniaturization with mild perifollicular fibrosis. **(D)** Occipital scalp, transverse section, showing reduced follicular density and increased miniaturized follicles (hematoxylin and eosin, × 5).

To objectively confirm occipital involvement and reduce the risk of donor-area misclassification, two synchronous 4-mm punch biopsies were obtained from the vertex and occiput. Specimens were processed with vertical and transverse sections. Key histopathologic variables included the terminal-to-vellus hair ratio, the proportion of miniaturized follicles, and the presence and severity of perifollicular fibrosis and inflammatory infiltrates graded on a 0 to 3 scale. Slides were reviewed independently by two dermatopathologists blinded to clinical information and sampling site, and any discrepancies were resolved by consensus. Histopathology supported pan-scalp miniaturization in both patients, with abnormal terminal-to-vellus ratios and increased miniaturized follicles in both the vertex and occiput. Vertex involvement was again more pronounced than occipital involvement, while mild perifollicular fibrosis and inflammation were observed in selected sites (Figure 3).

### 2.4 Therapeutic intervention

Both patients were counseled that, when occipital follicles demonstrate miniaturization, donor stability is uncertain and hair transplantation is generally discouraged until disease activity is clarified and stabilized. Both elected medical management. Oral finasteride (1.0 mg once daily) was prescribed as standard medical therapy for male AGA-spectrum disease. Topical minoxidil 5% was selected as an adjunct because it can be applied broadly to all clinically involved zones, including the occiput when affected, while maintaining a favorable systemic safety profile. Oral minoxidil may be a reasonable alternative in diffuse disease, particularly when large surface areas are involved, topical intolerance occurs, or adherence is poor. However, it requires individualized cardiovascular risk assessment and monitoring. For these reasons, topical therapy was used as first-line adjunctive treatment in this series. Both patients reported good adherence without major treatment interruption duringfollow-up.

### 2.5 Follow-up and outcomes

Follow-up was assessed at 6 months using a standardized clinic protocol. To improve longitudinal measurement consistency, target imaging areas were localized to the same anatomical positions at each visit and documented using high-resolution scalp photography, fixed anatomical landmarks, and skin marking when needed.

At 6 months, both patients showed objective improvement or stabilization. Non-vellus hair density increased and trichoscopic miniaturization decreased in both the vertex and occiput in each case, consistent with short-term stabilization and partial improvement on medical therapy. Both patients also reported reduced daily shedding and subjective stabilization of hair volume. In Case 1, an additional visit at 12 months suggested continued stabilization with partial trichoscopic improvement.

No procedure-related complications from scalp biopsy were reported. No new safety concerns were documented during medical therapy within the available follow-up period.

## 3 Discussion

These cases illustrate a clinically important pitfall in diffuse non-scarring alopecia: when the occiput is assumed to be a stable reference region, DUPA may be overlooked and donor-area counseling may be inaccurate. In classic AGA, miniaturization is usually concentrated in androgen-dependent regions with relative occipital preservation. In DUPA, by contrast, miniaturization may extend into the occipital scalp and other potential donor zones, undermining the concept of a reliably stable donor area for transplantation. Our approach combines two complementary assessments. First, paired vertex-occipital trichoscopy quantifies hair-shaft diameter variability and miniaturization in a non-invasive manner and can help distinguish DUPA from diffuse mimickers such as chronic telogen effluvium and alopecia areata incognita (7, 8). Second, paired biopsies provide objective confirmation of pan-scalp miniaturization by demonstrating abnormal terminal-to-vellus ratios and increased proportions of miniaturized follicles at both sites. In both patients, occipital involvement was clearly documented clinically, trichoscopically, and histopathologically. Standardized follow-up imaging further demonstrated short-term stabilization and partial improvement on medical therapy, supporting the value of quantitative monitoring in diffuse disease. From a surgical perspective, objective occipital miniaturization has immediate implications for donor selection. Hair transplantation depends on a relatively stable donor pool. When donor follicles are miniaturizing, harvesting may worsen visible thinning in the donor area and transplanted follicles may continue to miniaturize over time, resulting in unpredictable or unsatisfactory outcomes (9). Accordingly, hair transplantation should be approached with great caution and is often deferred in patients with confirmed occipital involvement consistent with DUPA. Nevertheless, in carefully selected and highly motivated patients, a cautious staged strategy may be discussed after sustained stabilization on medical therapy, supported by repeat trichoscopy demonstrating donor stability. Such an approach may include conservative graft numbers, avoidance of aggressive harvesting, consideration of a limited test session, and explicit counseling that continued pharmacotherapy and long-term follow-up are essential.

The strengths of this report include synchronous vertex and occipital assessment, the combination of quantitative non-invasive evaluation with histopathologic confirmation, and standardized follow-up using repeatable target localization. The main limitations are the small sample size, retrospective data collection, and the possibility of inter-operator variability in trichoscopic interpretation and image acquisition. Prospective studies are needed to determine how often occipital involvement is missed during routine evaluation, whether specific trichoscopic or pathologic features predict therapeutic response, and how quantitative donor metrics may inform surgical decision-making.

In summary, DUPA should be considered when thinning is relatively uniform across the scalp and the occipital region is not clearly spared. Occipital trichoscopy should be performed in all men evaluated for AGA, particularly when procedural options are being considered, to detect and monitor donor-area miniaturization. Paired vertex and occipital biopsies can be reserved for situations in which diagnostic certainty is likely to change management. When occipital miniaturization is present, clinicians should optimize medical therapy, monitor objectively over time, and approach surgical options with caution.

## Data Availability

The raw data supporting the conclusions of this article will be made available by the authors, without undue reservation.
